# Otitis media: recent advances in otitis media vaccine development and model systems

**DOI:** 10.3389/fmicb.2024.1345027

**Published:** 2024-01-24

**Authors:** Ayesha Zahid, Jennifer C. Wilson, I. Darren Grice, Ian R. Peak

**Affiliations:** ^1^Institute for Glycomics, Griffith University, Gold Coast, QLD, Australia; ^2^School of Pharmacy and Medical Science, Griffith University, Gold Coast, QLD, Australia

**Keywords:** otitis media, childhood disease, *Streptococcus pneumoniae*, *Haemophilus influenzae*, *Moraxella catarrhalis*, hearing loss, vaccine, animal models

## Abstract

Otitis media is an inflammatory disorder of the middle ear caused by airways-associated bacterial or viral infections. It is one of the most common childhood infections as globally more than 80% of children are diagnosed with acute otitis media by 3 years of age and it is a common reason for doctor’s visits, antibiotics prescriptions, and surgery among children. Otitis media is a multifactorial disease with various genetic, immunologic, infectious, and environmental factors predisposing children to develop ear infections. *Streptococcus pneumoniae, Haemophilus influenzae,* and *Moraxella catarrhalis* are the most common culprits responsible for acute otitis media. Despite the massive global disease burden, the pathogenesis of otitis media is still unclear and requires extensive future research. Antibiotics are the preferred treatment to cure middle ear infections, however, the antimicrobial resistance rate of common middle ear pathogens has increased considerably over the years. At present, pneumococcal and influenza vaccines are administered as a preventive measure against otitis media, nevertheless, these vaccines are only beneficial in preventing carriage and/or disease caused by vaccine serotypes. Otitis media caused by non-vaccine serotype pneumococci, non-typeable *H. influenza,* and *M. catarrhalis* remain an important healthcare burden. The development of multi-species vaccines is an arduous process but is required to reduce the global burden of this disease. Many novel vaccines against *S. pneumoniae,* non-typeable *H. influenza,* and *M. catarrhalis* are in preclinical trials. It is anticipated that these vaccines will lower the disease burden and provide better protection against otitis media. To study disease pathology the rat, mouse, and chinchilla are commonly used to induce experimental acute otitis media to test new therapeutics, including antibiotics and vaccines. Each of these models has its advantages and disadvantages, yet there is still a need to develop an improved animal model providing a better correlated mechanistic understanding of human middle ear infections, thereby underpinning the development of more effective otitis media therapeutics. This review provides an updated summary of current vaccines against otitis media, various animal models of otitis media, their limitations, and some future insights in this field providing a springboard in the development of new animal models and novel vaccines for otitis media.

## 1 Introduction

Otitis media (OM) is inflammation within the middle ear (ME), most frequently occurring in children. OM is caused by a variety of factors including bacterial or viral infections, allergy, Eustachian tube (ET) malfunction, physiological/immunological/pathological factors within the ME, as well as genetic and environmental factors (Yeo, 2018). Clinical manifestations of OM vary, depending on the type of exudate and the duration of the disease (Lieberthal et al., 2013). Acute OM (AOM) is the usual symptomatic presentation caused by an ongoing viral or bacterial infection accompanying fever, lethargy, otorrhea, irritability, vomiting, diarrhea, and in severe cases hearing loss. There is often a connection between AOM, and recent upper respiratory tract infections (URTI) caused by bacteria or viruses, in part because the ME is contiguous with URT via the ET. The less-developed ET/ME anatomy of young children is suggested as one factor that predisposes them to AOM (Toll and Nunez, 2012). ME disease recurs in many children, termed recurrent AOM (rAOM) (Granath, 2017). Some children are prone to develop AOM mainly due to immune dysfunctions. If they develop 4 AOM episodes in 6 months or 4 episodes in 12 months period, they are defined as otitis-prone children (Alho et al., 1991; Pichichero, 2020). Non-purulent fluid accumulation behind the tympanic membrane is a sign of OM with effusion (OME) which is often not associated with pain and may occur following AOM, rAOM, or other upper respiratory infections (Rosenfeld et al., 2016). OME makes children more prone to bacterial and viral infections. If it persists for extended periods, resulting in chronic OM (COM) (Granath, 2017), it can result in damage to the ME and conductive hearing loss. This is especially important for preschool children whose speech development may be delayed by this condition (Simon et al., 2018). The typical characteristics of COM are ear pain, ear discharge, and hearing impairment which may persist even after surgery, leading to many restrictions in daily life (Lucidi et al., 2022). Pro-inflammatory cytokines like TNF-α and IL-1, produced to combat bacterial infection during an AOM episode, set up an inflammatory response leading to the evolution of COM, which may include epithelial remodeling (Juhn et al., 2008). However, the exact mechanisms leading to COM are still poorly understood. Chronic suppurative OM (CSOM) is characterized by persistent or recurrent ear drainage (otorrhoea) for more than 2–6 weeks. During CSOM, bacterial pathogens may also infect the mucosa of the ME through the external canal (Jensen et al., 2017).

It is estimated that nearly 80% of young children (6–24 months old) suffer from at least one episode of OM each year (Tong et al., 2018). Its high prevalence in infants and children, despite low associated mortality, makes it a major health burden worldwide as it poses a substantial impact on the development of children, their families, and the health care system. OM is a frequent cause of visiting doctors, antibiotic prescriptions, and surgery among children (Coker et al., 2010) and the annual cost of OM to the healthcare system has been estimated to be more than $5 billion in the United States (Rovers, 2008) and $100 – $400 million in Australia (Kong and Coates, 2009). According to the World Health Organization (WHO), nearly 28 thousand people die due to OM globally, and nearly 50% of permanent hearing loss cases are caused by OM (Acuin, 2004). Indigenous populations across the world are at high risk for OM including Native Americans, the Alaskan, Canadian and Greenland Inuit, and Australian Aborigines (Bluestone, 1998; Daly et al., 2010).

**Figure 1:**
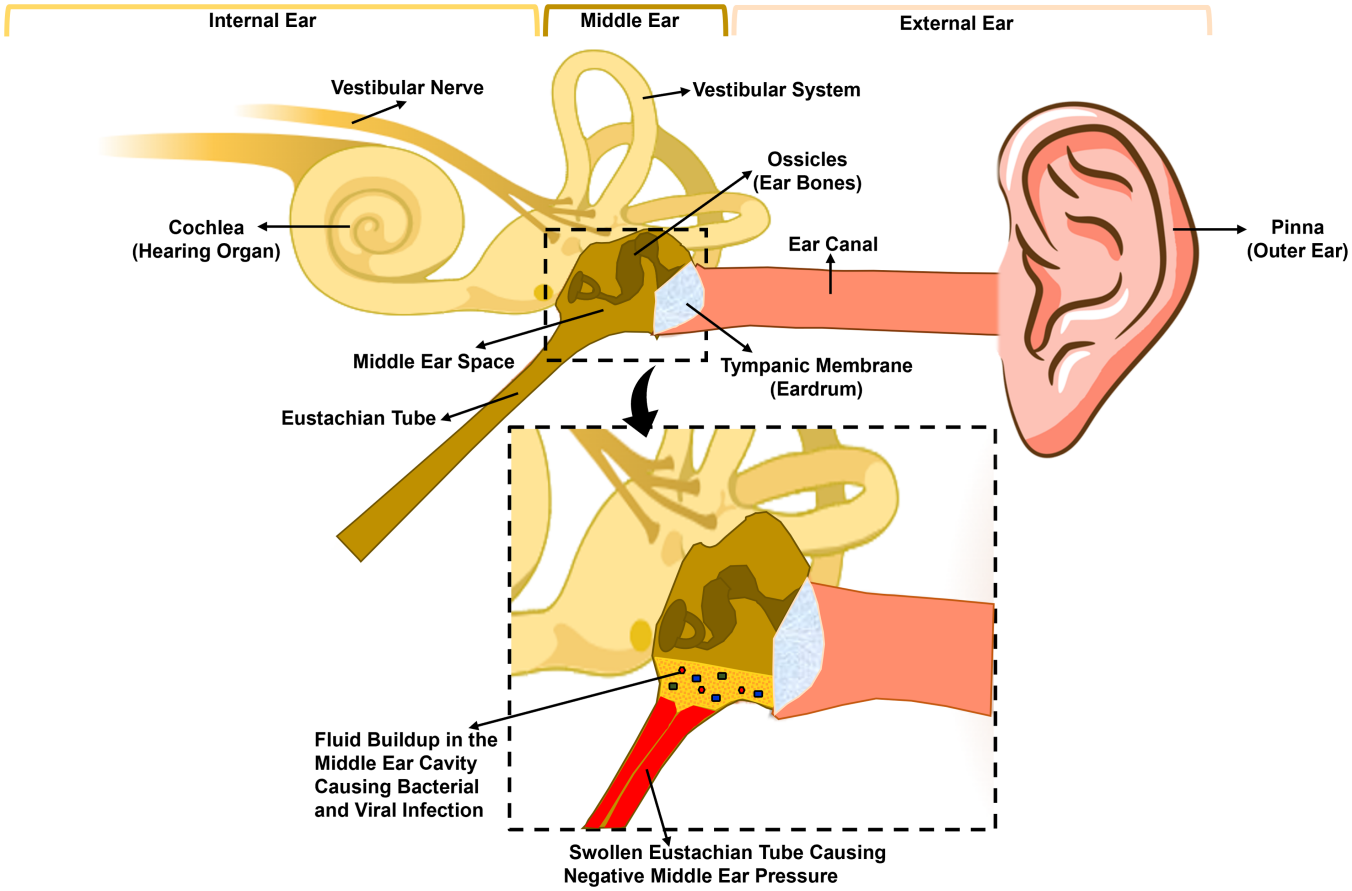
Anatomy of the human ear and pathogenesis of acute otitis media. The human ear is composed of three parts: the outer ear, the middle ear, and the inner ear. The tympanic membrane (eardrum) separates the outer ear, from the middle ear. The middle ear is composed of the middle ear cavity and the middle ear bones, which are attached to the tympanic membrane. The Eustachian tube connects the middle ear cavity to the nasopharynx. A viral or bacterial upper respiratory tract infection initiates inflammation of the nasopharynx and the Eustachian tube leading to the blockage of the latter trapping fluid inside the middle ear, leading to increased adherence and colonization of bacteria. Eustachian tube dysfunction also leads to negative middle ear pressure, allowing bacteria and/or viruses in the nasopharynx to move into the middle ear causing infection and inflammation.

The etiology of OM is often polymicrobial where the most common bacterial pathogens associated with AOM are *Streptococcus pneumoniae* (*S. pneumoniae*), non-typeable *Haemophilus influenzae* (NTHi), and *Moraxella catarrhalis* (Mcat). Contribution to disease by each pathogen varies by study and detection method [see (Tamir et al., 2023] for review of recent reports of microbiology of OM). The viruses that are commonly associated with OM include respiratory syncytial virus (RSV), coronaviruses, influenza viruses, adenoviruses, human metapneumovirus, human bocavirus, and picornaviruses (Pettigrew et al., 2011; Ngo et al., 2022). The most commonly associated pathogens with CSOM are *Staphylococcus aureus* and *Pseudomonas aeruginosa* (Afolabi et al., 2012; Khattak et al., 2017). Fungi and anaerobic bacteria have also been associated with OM/CSOM (Prakash et al., 2013). The anaerobic bacteria associated with OM/CSOM are *Prevotella melaninogenica, Clostridium* spp., *Peptococcus* spp., and *Fusobacterium* spp. (Brook, 2008). Bacteria that are commonly associated with OME are coagulase-negative staphylococci, *Veillonella* spp., and *S. aureus* (Daniel et al., 2012). Viral infections predispose the host to bacterial infections of the ME, particularly influenza A virus, coronavirus, and respiratory syncytial virus (RSV) [reviewed by (Marom et al., 2012)]. For the development of OM when the tympanic membrane is intact, the pathogen needs to establish nasopharyngeal colonization by gaining access to the tympanic cavity via the ET, as illustrated in Figure 1.

The ME mucosa, like other mucosal surfaces in the body, has a local immune system that plays a critical role in protecting against infections. The ME mucosa and URT are connected anatomically, and their immune systems are interconnected as well (Massa et al., 2021; Kurono, 2022). To prevent OM, enhancement of mucosal immune responses in the nasopharynx (NP) and ME is desired, however, how the immune protection in the ME mucosa relates to URT is still poorly understood.

Antibiotics are frequently used as a treatment strategy for OM, nonetheless, high morbidity and the alarming rise in antibiotic resistance over the years have increased interest in the development of alternative strategies such as vaccination. The development of several vaccines has been achieved to combat disease caused by *S. pneumoniae*, such as a 14-valent pneumococcal polysaccharide vaccine (1977), the 23-valent pneumococcal polysaccharide vaccine (1983), a 7-valent pneumococcal conjugate vaccine (2000), and a 13-valent pneumococcal conjugate vaccine (2010) (Grabenstein and Klugman, 2012). Although these vaccines are effective against OM, they do however present the risk of further serotype replacement (Weinberger et al., 2011; Lo et al., 2019). Pneumococcal vaccine development has received priority because pneumococcus causes life-threatening pneumonia and invasive diseases, including sepsis and meningitis. Vaccines against NTHi and Mcat, however, are not currently available, despite both species causing significant morbidity through OM, pneumonia, exacerbations of chronic lung disease, and other diseases of respiratory mucosa in adults and children. Advances have been made in the development of vaccines targeting the major otopathogens, and many potential vaccine antigens from these bacteria have been investigated. This review aims to offer an updated summary of candidate vaccine antigens, including their putative or known functions. Furthermore, it also discusses currently used animal models of OM, which are crucial for relevant pre-clinical assessment of candidate antigens and vaccine formulations.

## 2 Vaccines against OM

An ideal vaccine antigen will possess some essential features leading to broad protection against the target pathogen. First, the vaccine antigen needs to be conserved among clinically relevant strains so that it can provide the necessary coverage (Murphy, 2005). The majority of bacterial otopathogens are highly adapted to the colonization of the human URT and are frequent asymptomatic colonizers. As such, surface-exposed regions are under immune-pressure. Many surface-exposed epitopes have variable sequences between strains or are expressed at different levels between strains, and this inter-strain variability needs to be known (Karls and Perkins-Balding, 2013). If a targeted gene is not present in all strains, or it is antigenically variable between strains, then one or more additional vaccine candidates may be required for optimal protection. The development of multi-protein vaccines is becoming increasingly recognized as necessary for pathogens with highly variable antigens between and within strains (Watson et al., 2019). However, such formulations can lead to antigenic competition, or masking of key antigenic epitopes leading to compromised immune responses to one or more antigens (Michel et al., 2022). This requires analysis of antigens and responses in animal and human trials.

Secondly, it is highly desirable that the antigen is exposed on the surface of the pathogen so that this region can be recognized by the antibodies. Ideally, the antigen is essential for colonization or pathogenesis, and its function is known (Murphy, 2005). Moreover, if the candidate vaccine has some toxicity, genetic or chemical modification will be required (Rappuoli, 1999). Importantly, it is necessary to establish if there is a risk of autoimmunity, e.g., if there is significant sequence or structural similarity to host antigens (Kraus et al., 1989). Most importantly, the vaccine antigen needs to be immunogenic in the host and elicit a lasting protective immune response upon immunization, induced by humoral or cellular immunity (Murphy, 2005).

During the last few decades, both computational and experimental methods have been utilized to identify potential vaccine antigens for major pathogens involved in OM. In this review, we focus on those potential antigens that have progressed substantially as vaccine candidates and show potential for inclusion in vaccines that will reduce the burden of OM.

### 2.1 Pneumococcal vaccines

#### 2.1.1 Pneumococcal conjugate vaccines (PCVs), pneumococcal polysaccharide vaccine (PPV), and OM

*Streptococcus pneumoniae* is naturally found in the human NP, and this nasopharyngeal carriage is important for active infection and transmission of the pathogen (McDaniel and Swiatlo, 2004). Its capsular polysaccharides (CPSs) are important virulence factors, especially for invasive disease that can be classified into 100 serotypes, based on chemical and immunological profiles (Ganaie et al., 2020). The most identified pathogen in OM following culture is *S. pneumoniae* (Tamir et al., 2023).

Currently, two types of pneumococcal vaccines are available. Both are based on CPS: (A) the 23-valent pneumococcal polysaccharide-based vaccine (PPV-23); and (B) the 7-, 10-, 13-, and 20-valent pneumococcal conjugate vaccines (PCV-7, −10, −13, −20).

PPV23 is based on the CPS antigens from 23 different serotypes of *S. pneumoniae* and is recommended for individuals aged 65 and older, as well as those aged 2 to 64 years who have multiple chronic conditions, such as diabetes and chronic cardiovascular disease (CDC, 2019). Most bacterial CPS are poorly immunogenic in young children, in part because they are T-cell independent antigens and induce little immune memory. Newborns and infants up to the age of 1.5–2 years of age are unable to produce antibodies to bacterial CPS due to less developed immunity (Rijkers et al., 1998), therefore, there is much debate surrounding the use of pneumococcal polysaccharide vaccines in young children (Borrow et al., 2012). In PCVs, the pneumococcal polysaccharide is coupled to a carrier protein to convert the antigens from T-cell independent to T-cell dependent antigens for enhanced immunogenicity (Pichichero, 2013). PCVs have been incorporated into the national immunization programs of 148 out of 194 World Health Organization (WHO) member states as of the end of 2020 (CDC, 2020). There has been a decline in OM and NP colonization by vaccine serotypes of *S. pneumoniae* with these vaccines, however, their use has been linked with replacement by non-vaccine serotypes of *S. pneumoniae* as well as NTHi and Mcat (Barenkamp et al., 2017; Kaur et al., 2017; Ben-Shimol et al., 2019; Pereira et al., 2023). Pneumococcal serotype replacement after vaccination has been noted for both childhood carriage (Lee et al., 2017) and in AOM (Gene et al., 2013), and in some cases the replacing strains are antibiotic resistant (Lo et al., 2019), so that vaccination may change the distribution of resistance and potential treatment failures not only for URTI/OM but also for pneumonia or invasive disease. A study in Israel evaluated the efficacy of PCV7 and PCV13 and reported that these vaccines reduced pneumococcal AOM by 77%, however, an increase in AOM cases due to non-vaccine serotypes was observed (Ben-Shimol et al., 2014). Similarly, a Costa Rican study of AOM in children reported that pneumococcus had a lower frequency and NTHi cultured at a higher frequency in vaccinated vs. unvaccinated children (Abdelnour et al., 2015). A 14-year observational study on the effects of PCV7, PCV10, and PCV13 in Swedish preschool children showed that Mcat and NTHi were proportionally more common than *S. pneumoniae* after PCV introduction in NP culture of PCV-immunized children with URTI (Littorin et al., 2021). A global surveillance study that used whole-genome sequencing for analysis reported that non-PCV serotypes15B and 15C (15B/C), 12F, and 35B/D were among the most prevalent pneumococcal serotypes in the post-PCV13 era (Lo et al., 2019). In Italy, non-vaccine serotype 24F has been reported as a prevalent serotype in pneumococcal otorrhea cases (Sings et al., 2019). Such epidemiological studies of pneumococcal serotypes from both carriage and OM inform future changes to polyvalent vaccines. Notably, PCV-20 includes several recently emergent serotypes (1, 3, 4, 5, 6A, 6B, 7F, 8, 9 V, 10A, 11A, 12F, 14, 15B, 18C, 19A, 19F, 22F, 23F, and 33F) and is now approved for use in all age groups in the United States (ACIP, 2023).

As there are at least 100 pneumococcus serotypes, increasing the number of vaccine serotypes in the PCV vaccines is more complex and expensive. In summary, both PPV and PCVs induce serotype-specific immunity and their usage has been linked with the emergence of replacement serotypes (Weinberger et al., 2011; McElligott et al., 2015; Kaur et al., 2016; Kawaguchiya et al., 2017). Nonetheless, the success of PCVs in reducing childhood mortality and morbidity due to pneumonia and invasive disease is significant, for example, see a recent review of pneumococcal disease in children in the United States (Walter and Smith, 2022).

#### 2.1.2 Protein-based pneumococcal vaccines in clinical and pre-clinical trials

Despite the success of PCVs, the challenge of regular re-formulation, and the theoretical and practical challenges of including more serotypes means the search for protein antigens has continued, aimed at developing pneumococcal vaccines targeting conserved and universally present pneumococcal proteins to complement or ultimately substitute PCVs. A protein-based vaccine would theoretically be simpler and cheaper to make than a conjugate vaccine. Several protein antigens have been investigated in human trials, individually, and in multivalent formulations. Table 1 summarizes antigens and vaccine candidates for pneumococcal diseases [Also recently reviewed by (Duke and Avci, 2023)].

**Table 1:** Potential vaccine antigens of *Streptococcus pneumoniae* at various stages of development.

Antigen(s)	Putative antigen function/other features	Adjuvant	Current development stage	References
PhtD	Histidine-triad protein D	Aluminum hydroxide, aluminium phosphate, AS02	Phase 1 completed	Brookes et al. (2015)
PlyD1	Cholesterol-dependent toxin	Aluminum hydroxide	Phase 1 completed	Kamtchoua et al. (2013)
PlyD1 + PcpA + PhtD	A trivalent vaccine containing Ply, PcpA, and PhtD	Aluminum hydroxide	Phase 1 completed	Brookes et al. (2015) and Ren et al. (2015)
PHiD-CV/dPly/PhtD-10/30	Vaccine formulations containing pneumolysin toxoid (dPly) and PhtD each at either 10 μg (PHiD-CV/dPly/PhtD-10) or 30 μg (PHiD-CV/dPly/PhtD-30).	Aluminum phosphate	Phase 1 and Phase 1/2 safety and immunogenicity completed, Phase 2b infants vs. AOM, Phase 2 carriage and non-inferiority to PCV-10	Leroux-Roels et al. (2014), Prymula et al. (2014), Odutola et al. (2016, 2017), and Prymula et al. (2016)
PcpA, PcpA + PhtD	Monovalent, and bivalent vaccine containing choline-binding protein A and PhtD	Aluminum hydroxide	Phase 1 completed	Bologa et al. (2012)
wSp	*Streptococcus pneumoniae* whole-cell vaccine	Aluminum hydroxide	Phase 1 and 2 completed	NCT02543892 (2018) and Keech et al. (2020)
PnuBioVax	Detergent extract of TIGR4 strain with non-toxic Ply	None	Phase 1 completed (adults)	Entwisle et al. (2017)
*Salmonella Typhi* expressing PspA	A live vector expressing pneumococcal surface protein A	None	Phase 1 (dose-escalation trial) completed	Frey et al. (2013)
PsaA	Pneumococcal surface adhesin A involved in bacterial adherence and virulence	Aluminum hydroxide, cholera toxin (CT), nanogel based delivery system, bacterium-like particle (BLP) delivery system	Phase 1 completed	Briles et al. (2000a), Kong et al. (2013), González-Miro et al. (2017), and Wang et al. (2018)
PspA+PlyD	A bivalent vaccine containing PspA and PlyD	Not available	Phase 1a completed	NCT04087460 (2022)
CpbA+ L460D pneumolysoid	A fusion protein consisting of the choline binding protein A peptide and L460D, a nontoxic pneumolysoid	Alum	Animal studies	Mann et al. (2014)
MAV	A multiple-antigen vaccine (MAV) prepared from *Streptococcus pneumoniae* TIGR4 lysates	None	Animal studies	Chan et al. (2022)
Elongation factor Tu (EF-Tu)	Surface protein involved in the catalysis of aminoacyl-tRNA binding to the ribosome, inhibits protein synthesis	FCA	Animal studies	Nagai et al. (2019)
LytB	A murein hydrolase involved in nasopharyngeal colonization and cell separation	Aluminum hydroxide	Animal studies	Ren et al. (2015) and Corsini et al. (2016)
PspAB_1-5_	A recombinant PspA-based protein vaccine consisting of the B region fragments from clades 1 to 5 of both families 1 and 2	Alum	Animal studies	Akbari et al. (2019)
Pneumococcal Δ*pep27ΔcomD* whole cell vaccine	Whole-cell pneumococcus lacking *pep27* and *comD* genes	None	Animal studies	Kim et al. (2019)
Pneumococcal TIGR4*Δlgt* whole cell vaccine	A whole-cell pneumococcal vaccine based on *lgt*-deficient TIGR4 strain	None	Animal studies	Jang et al. (2019)

BLP, bacterium-like particle; CpbA, choline-binding protein A; dPly, detoxified pneumolysin; EF-Tu, elongation factor Tu; FCA, Freund’s complete adjuvant; L460D, non-toxic pneumolysoid with the L460D substitution; LytB, an endo-β-N acetylglucosaminidase; lgt, prolipoprotein diacylglycerol transferase gene; NA, not available; OM, otitis media; PlyD1, detoxified pneumolysin derivative; PhtD, polyhistidine triad protein D; PhtE, polyhistidine triad protein E; PsaA, pneumococcal surface adhesin A; PcpA, pneumococcal choline binding protein A; PHiD, non-typeable *Haemophilus influenzae* protein D; PCV, pneumococcal conjugate vaccine; wSp, *Streptococcus pneumoniae* whole cell vaccine; WCA, whole cell antigen; μg, microgram.

Pneumolysin (Ply) is a cholesterol-dependent cytolysin that has haemolytic properties, and aids in bacterial pathogenesis and infection. Vaccination with a detoxified or non-toxic form of Ply (dPly) is known to confer protection against multiple serotypes in animal models (Alexander et al., 1994; Ogunniyi et al., 2001; Denoël et al., 2011). A genetically mutated pneumolysin derivative (PlyD1) was safe, robustly immunogenic, and induced neutralizing antibody responses in a phase 1 trial (NCT01444352) (Kamtchoua et al., 2013). More recently, a genetic toxoid of Ply with two amino acid mutations (G293S and L460D) termed Ply-D protected mice from the lethal challenge of various clinical pneumococcal isolates (Thanawastien et al., 2021).

Pneumococcal histidine triad protein D (PhtD) is a highly conserved adhesin protein. The PhtD-based vaccines protect immunized mice against NP and lung colonization of pneumococcus (Khan and Pichichero, 2012; Kaur et al., 2014b; Brookes et al., 2015; Ochs et al., 2016). Combining PlyD1 and PhtD, and including PHiD-10 (conjugated to protein D of *H. influenzae*), Odutola et al. (2016, 2017, 2019) reported on phase 2 trials in African children (NCT01262872), while the same vaccine was assessed in toddlers in Czech republic for safety, immunogenicity, and non-inferiority to PCV-10 (Urbancikova et al., 2017). None of these trials assessed protection against OM. Pneumococcal surface protein A (PspA) is a choline-binding protein that is located on the cell surface in approximately all pneumococcus strains and is involved in the complement-mediated clearance of bacteria. PspA was shown to be safe during phase I clinical trials (Briles et al., 2000b). However, PspA shows homology to the human cardiac myosin and must be modified to prevent possible autoimmune disease or cardiac inflammation (Ginsburg et al., 2012). PspA recently underwent phase 1a clinical trial as part of a multi-element vaccine alongside PlyD (NCT04087460, 2022). Pneumococcal choline-binding protein A (PcpA) is a surface-exposed protein that is conserved among different pneumococcal strains (Glover et al., 2008). A bivalent PcpA/PhtD vaccine was immunogenic and safe in humans during a phase 1 clinical study (NCT01444339) (Bologa et al., 2012), and induced functionally relevant antibodies (Ochs et al., 2016). A trivalent vaccine containing recombinant PcpA, PhtD, and PlyD1 (designed from serotype 6B) protected infant mice from the serotype 6A challenge (Verhoeven et al., 2014). This vaccine formulation was considered safe and immunogenic in a phase 1 study including adults, toddlers, and infants (NCT01764126) (Brookes et al., 2015). Furthermore, serum IgG antibody responses against PhtD, PcpA, and PlyD1 were in synchrony, indicating they are similarly immunogenic and therefore compatible with combining in a trivalent protein vaccine (Ren et al., 2015).

Another serotype-independent pneumococcal protein vaccine candidate called PnuBioVax™ is expected to offer broader coverage at a low unit price. This vaccine is formulated by fermenting a genetically modified *S. pneumoniae* TIGR cell substrate. Following harvesting, the protein antigens are detergent-extracted and purified using ion exchange chromatography (Cecchini et al., 2015). During phase 1 human trials (NCT02572635), this vaccine demonstrated good safety and immunogenicity profile (Entwisle et al., 2017; Hill et al., 2018).

Other antigens have been investigated in animal models and show promise for inclusion in a multivalent protein vaccine. Kong et al. developed a PspA vaccine with an intranasal vaccine delivery system based on a nanometre-sized hydrogel (nanogel) composed of a cationic cholesteryl group-bearing pullulan (cCHP). Intranasal vaccination with the cCHP-PspA vaccine protected mice from a lethal *S. pneumoniae* Xen10 challenge by reducing the invasion and colonization of bacteria in the upper and lower respiratory tracts (Kong et al., 2013). Wang et al. utilized a bacterium-like particle (BLP) delivery system designed to express PspA on the BLP surface. Intranasal delivery of this vaccine-induced PcpA-specific IgG and IgA antibodies in mice and protected the animals from a lethal challenge with *S. pneumoniae* (Wang et al., 2018). A recombinant PspA-based protein vaccine (PspAB_1-5_) consisting of the B region fragments from clades 1 to 5 of both families resulted in improved C3 complement component depositioning in immunized mice. The antibodies induced were cross-reactive against pneumococci from clades 1, 2, and 5. Consequently, the PspA vaccines based on the B region of all clades may be able to provide better protection against *S. pneumoniae* (Akbari et al., 2019).

Elongation factor Tu (EF-Tu) is a universally expressed surface protein that is highly conserved in different *S. pneumoniae.* EF-Tu has chaperone activity and mediates peptide biosynthesis, protein folding, and cellular stress response (Gregersen and Bross, 2010). A vaccine containing recombinant EF-Tu from *S. pneumoniae* strain D39 protected immunized mice against lethal challenges with serotype 2 and a multidrug-resistant serotype 15A, and increased the levels of cytokines including TNFα, IL-6, IL-17, and IFN-γ. The anti-EF-Tu serum demonstrated an enhanced phagocytic activity against *S. pneumoniae*, irrespective of its serotypes (Nagai et al., 2019). Hence, EF-Tu presents as a potential broad-spectrum vaccine candidate against common pneumococcal serotypes. LytB is an endo-β-N acetylglucosaminidase involved in the formation of biofilm, separation of daughter cells, and pathogenesis (Bai et al., 2014). An anti-LytB antiserum demonstrated significant protection in mice from a lethal pneumococcal challenge (Wizemann et al., 2001). In another study mice immunized with LytB showed enhanced complement-mediated immunity against various pneumococcal serotypes. Anti-LytB serum-stimulated neutrophil-mediated phagocytosis against *S. pneumoniae* (Corsini et al., 2016). Thus, LytB could be an effective antigen in a pneumococcus vaccine.

In an interesting approach, Mann et al. fused peptides of choline-binding protein A (CbpA) with a non-toxic Ply (L460D). In mice, this fusion was more effective than L460D at reducing nasal colonization, OM, pneumonia, OM, and meningitis (Mann et al., 2014).

#### 2.1.3 Live vector based pneumococcal vaccine

Shi et al. developed three live recombinant *Salmonella Typhi* vectors expressing PspA pneumococcal protein. These live vectors were shown to be highly immunogenic in mice and highly susceptible to killing in human blood (Shi et al., 2010). In a phase I clinical trial, administration of these live attenuated Salmonella strains was safe and well-tolerated in healthy adult subjects. The immunogenicity profile of these live vectors was limited perhaps because of pre-existing cross-reactive antibodies, therefore further genetic manipulation is needed to develop a candidate with improved immunogenicity (Frey et al., 2013).

#### 2.1.4 Whole-cell vaccines

Whole-cell vaccines are an attractive alternative to polysaccharide-based vaccines because they take advantage of whole-cells expressing various protein antigens without involving the purification of individual antigens. Live-attenuated or killed whole-cell vaccines from unencapsulated *S. pneumoniae* can provide serotype-independent protection in animal models. One caveat is that use of live attenuated strains must be used cautiously in populations with a risk of impaired or sub-optimal immune status. The pneumococcal whole-cell vaccine (wSp) consists of a killed, unencapsulated pneumococcal strain delivered with an alum-based adjuvant. The purpose was to provide a cost-effective vaccine with broader coverage. In phase 1 studies in healthy adults in the United States (NCT01537185), the vaccine demonstrated a good safety, tolerability, and immunogenicity profile (Keech et al., 2020). It has also been tested in phase 1 and 2 trials in healthy adults and toddlers in Kenya to evaluate its safety and immunogenicity which was reported to be satisfactory (NCT02543892). Although it has not been assessed for its efficacy in the human paediatric OM population, a subcutaneous dose of wSp decreased the density of pneumococcus in the ME of mice, however, it could not protect from *S. pneumoniae*-induced OM (Manning et al., 2019). A whole-cell vaccine consisting of an ethanol-killed capsule-deficient *S. pneumoniae* mutant prevented the colonization of serotype 19F and 4 strains in mice (Xu et al., 2014; Jang et al., 2019). Chan et al. reported a multiple-antigen vaccine (MAV) based on bacterial lysates. In addition to having the advantages of a whole-cell vaccine, the preparation of this vaccine improved the amount of surface antigens as it had heat shock proteins (Hsps), PspA, and Ply proteins. In animal studies, MAV was able to elicit functional antibody production against multiple serotypes of *S. pneumoniae*, including non-vaccine serotypes (Chan et al., 2022). Based on these observations, it can be anticipated that the MAV approach may confer serotype-independent protection against *S. pneumoniae*.

Immunization of mice with a live attenuated whole-cell vaccine based on *S. pneumoniae* D39 (lacking *pep27* and *comD* genes to eliminate the reversion of the bacterium to wild-type phenotype) showed significantly higher IgG titers against serotype D39. This immunization also reduced colonization regardless of the serotype. Furthermore, this strain depicted a good safety profile in both normal and immunocompromised mice. Thus, the *Δpep27ΔcomD* strain presents efficiency and safety in preventing pneumococcal infections (Kim et al., 2019). Another live attenuated vaccine strain has the pro-lipoprotein diacylglycerol transferase (*lgt*) gene deletion from the capsule of the pneumococcal strain TIGR4 (TIGR4*Δlgt*). This strain evokes a significantly reduced inflammatory response and is reduced in virulence. Intranasal immunization of mice resulted in protection against invasive pneumococcal infections (Jang et al., 2019). Thus, TIGR4*Δlgt* is an attractive broad-spectrum vaccine candidate.

### 2.2 NTHi vaccines against OM

NTHi commonly colonizes the URT and is predominantly present in the upper respiratory nasopharyngeal microbiota. It is associated with various non-invasive infections including OM, non-bacteremic pneumonia, and sinusitis (Van Eldere et al., 2014). NTHi is the main culprit of recurrent and chronic OM (Pichichero et al., 2008; Casey et al., 2010; Jalalvand and Riesbeck, 2018). As discussed earlier, PCVs have contributed to the increased proportion of OM associated with NTHi strains (Van Eldere et al., 2014). Prevention of NTHi-associated OM could reduce the global burden of OM by an estimated 350 million fewer annual cases of AOM episodes (Barenkamp, 2013). Since NTHi improves the NP colonization of Mcat (Andrade et al., 2018), therefore, to infect the ME and establish polymicrobial OM a symbiotic relationship between Mcat and NTHi is suspected (Armbruster et al., 2010). Given these observations, it can be anticipated that effective vaccine strategies specifically against NTHi-induced OM may also confer an indirect benefit for the prevention or resolution of polymicrobial OM.

The search for a safer broadly cross-reactive immuno-protective NTHi antigen has been expanding recently, however, to achieve this goal there are significant obstacles, mainly due to the heterogeneous nature of moieties present on the surface of NTHi: most surface-exposed antigens are variably present across strains, antigenically variable, phase variable, or a combination of more than one of these. At present, no NTHi-specific vaccines are licensed and the only NTHi antigen incorporated in a licensed vaccine is protein D as the protein conjugate in Synflorix™ (PHiD-CV). PhiD-CV is also in experimental vaccines with other NTHi antigens. Both PHiD-CV and PCV-10 likely reduce all-cause OM, but there is not strong evidence that Protein D in this formulation reduces carriage of NTHi or OM disease caused by NTHi (de Sévaux et al., 2020; Beissbarth et al., 2021). Given these observations and the expansive heterogeneity of NTHi strains, a multicomponent vaccine is essential to confer adequate protection against NTHi (Price et al., 2015; Jalalvand and Riesbeck, 2018).

#### 2.2.1 Recent vaccine candidates against NTHi

Numerous NTHi vaccine candidates have been investigated in the past years. In this section, we review only those candidates who presented great potential to undergo further development.

Many NTHi proteins including *H. influenzae* adhesin protein (Hap), *H. influenzae* autotransporter (Hia), Protein D (PD), outer-membrane protein 6 (P6), and outer-membrane protein 26 (OMP26) have been studied by several groups for the prevention of NTHi disease (see review by (Khan et al., 2016)). Antisera raised in guinea pigs against high molecular weight (HMW) proteins HMW1/HMW2 or Hia proteins showed opsonophagocytic activity against a wide range of NTHi strains (Winter and Barenkamp, 2014). Given these observations, a vaccine formulated with HMW1/HMW2 and Hia proteins may protect against a broader range of NTHi strains. Pre-clinical studies with these proteins revealed that PD immunization with OMP26 produced lowered antibody responses against PD, however, PD immunization with P6 did not mask PD immune responses (Michel et al., 2022), emphasizing the necessity of compatibility studies when combining antigens. Mice immunized with a truncated adhesin protein F (PF) showed faster clearance of NTHi infections compared to mice immunized with a control peptide (Jalalvand et al., 2014). Type IV pili (Tfp) are crucial for NTHi biofilm development, adherence, competence, and twitching motility (Das et al., 2017). Antisera raised against recombinant soluble PilA (rsPilA) dispersed *in vitro* formed NTHi biofilms and blocked NTHi-induced OM in chinchilla (Novotny et al., 2015b, 2017).

A recombinant fusion protein consisting of immunologically important components of protein E (PE) and PilA was evaluated in pre-clinical trials. PE-PilA-induced anti-PilA antibodies halted NTHi biofilm development and disrupted *in vitro* established biofilms, as seen for rsPilA. After the intranasal NTHi challenge, NP colonization was significantly reduced in immunized mice, and in chinchillas, symptoms of experimental OM were notably hindered (Ysebaert et al., 2019). A multi-component NTHi vaccine consisting of a free recombinant PD and a recombinant fusion protein PE-PilA was tested in a phase 1 clinical trial in healthy adults (NCT01657526), with a good safety profile and acceptable reactogenicity (Leroux-Roels et al., 2016). A further development added the Mcat antigen UspA2 to this combination, to test a multi-component vaccine in two-dose and three-dose phase 1 and 2 clinical trials, showing promising safety and immunogenicity profiles in healthy adults and adults aged 50–71 with a history of smoking (De Smedt et al., 2021; Galgani et al., 2022).

Other vaccine candidates have not yet been tested in human trials but show promise in a range of animal studies. Intracellular elongation factor thermal-unstable (EF-Tu) was identified recently as a novel NTHi surface protein (Thofte et al., 2018); and it was reported that antibodies against EF-Tu can result in complement-dependent killing of NTHi (Thofte et al., 2019), indicating the potential of this protein as a NTHi vaccine antigen. To date, no further investigations in animal models have been reported.

In a parallel approach to the recombinant PE-PilA, Novotny et al. found that ChimV4, a fusion of P5 peptide and rsPilA resolved NTHi-induced OM when delivered transcutaneously (Novotny et al., 2015a). The formulation also prevented OM in a viral-bacterial model of experimental disease. Transcutaneous immunization with chimV4 + dmLT significantly increased mature B-cell phenotypes and antibody-secreting cells within nasal-associated lymphoid tissues (Novotny et al., 2017; Novotny and Bakaletz, 2020). A bioinformatics approach to identify candidate peptides could result in more rapid development of polyvalent protein antigens (see section 2.2.5 on recombinant polypeptide antigens).

One challenge for mucosal pathogens is eliciting a response to target bacteria at the surfaces. Building on previous studies that showed NTHi P6 as a promising candidate (DeMaria et al., 1996; Bakaletz et al., 1999; Kodama et al., 2000;), Kodama et al. found that prior treatment with CCL20 enhanced murine responses and bacterial clearance following intranasal P6 immunization (Kodama et al., 2011a). Similarly, P6 was tested with FMS-like tyrosine kinase receptor 3 ligand as a mucosal adjuvant and this combination also improved nasopharyngeal clearance of NTHi and dendritic cell recruitment (Kodama et al., 2011b; Kodama and Suzuki, 2011). Nasal immunization with P6 protein alone produced minimal or no antigen-specific immune responses and hence no effective protection against NTHi (Bertot et al., 2004; Abe et al., 2006; Noda et al., 2011). More recently, when combined with cCHP, a cationic cholesteryl pullulan nanogel, cCHP-P6 nanogel nasal vaccine induced P6-specific mucosal IgA and serum IgG responses without additional biologically active adjuvant. The P6-specific IgG titers were equivalent to those generated by the intramuscularly administered vaccine containing alum adjuvant (Nakahashi-Ouchida et al., 2022). This highlights that route of delivery, and adjuvant choice are crucial to assess vaccines to reduce disease at mucosal surfaces.

#### 2.2.2 Lipo-oligosaccharides (LOS) as NTHi vaccine candidates

LOS are a major component of the Gram-negative bacterial outer membrane and thus, in theory, they are an attractive target for vaccine development. In NTHi, five different antigenically heterogeneous LOS serotypes I-V are produced (Campagnari et al., 1987), but these glycans are phase-variable within a strain and occur during infection (Fox et al., 2014). Despite this, several studies examined LOS as a candidate vaccine. In a mouse model of NP colonization, intranasal immunization with detoxified LOS-tetanus toxoid (*d*LOS-TT) demonstrated a substantial reduction of the same LOS type III NTHi strains as well as types IV and V, but only 50% reduction of type I and 29% reduction of type II NTHi strains (Hirano et al., 2003). Parenteral immunization with *d*LOS conjugates in chinchillas produced anti-LOS antibodies in serum or ME which were bactericidal and opsonophagocytic and lowered homologous NTHi-induced OM (Sun et al., 2000). Notably, *d*LOS was tested in a phase I study of healthy adults vaccinated intramuscularly with *d*LOS-TT resulting in increases in serum antibodies, with acceptable safety. However, no further human trials have proceeded. An alternate approach is to target a glycan present in bacteria but absent from host tissues. Ketodeoxyoctonic acid (KDO) is present in most lipo-polysaccharides (LPS)/LOS, as well as in some CPS. A monoclonal antibody, 6E4, raised against KDO is bactericidal against 12 out of 33 tested strains of NTHi *in vitro* (Apicella et al., 2018). Using a glycoconjugate composed of either multiple KD residues or a KDO-*N*-acetyl-lactosamine conjugated to an immunogenic protein may be an approach to develop a component of a vaccine that would target many bacterial species.

#### 2.2.3 Outer membrane vesicles (OMVs) as NTHi vaccine candidates

OMVs produced by Gram-negative bacteria are enriched in outer membrane components, including major and minor outer membrane proteins and LOS. The functional activity of NTHi OMV-specific antisera and the protective ability of NTHi OMVs as vaccine antigens in the chinchilla OM model were tested. Immunization of chinchillas with OMVs isolated from HMW1/HMW2- and Hia- Hia-expressing NTHi prevented experimental OM (Winter and Barenkamp, 2017). In another study, immunization of NTHi-derived OMVs along with CpG-MPLA adjuvant induced the production of protective antibodies and cytokines in mice (Behrouzi et al., 2020). Several licensed and effective vaccines targeting meningococcal disease are based on OMVs. The presence of immunodominant proteins can lead to strain-specific responses, but either the removal of strain-specific antigens or the addition of conserved antigens can address this problem (Pizza et al., 2020). This approach may be feasible for both NTHi and Mcat but requires significant development.

#### 2.2.4 Lipidated NTHi antigens

Adding a lipid moiety to a recombinant protein is expected to increase the immunogenicity through Toll-Like Receptor 2 (TLR2) signaling of antigen-presenting cells and T-helper 17 cells (Th17) to evoke or enhance cellular responses in the nasal-associated lymphoid tissue (NALT). Recently, the effects of lipidation vs. non-lipidation of recombinant P6 and OMP26 were compared in a mouse model. Lipidated P6 and OMP26 elicited nearly 10- to 100-fold higher IgG antibody levels, and lipidated antigens also reduced NP colonization and ME bullae NTHi density more than non-lipidated formulation (Kaur and Pichichero, 2022). Therefore, lipidation of NTHi antigens represents a promising approach in the development of novel NTHi vaccine formulations (Table 2).

**Table 2:** Potential vaccine antigens of non-typeable *Haemophilus influenzae* at various stages of development.

Antigen	Putative antigen function/other features	Adjuvant	Current development stage	References
Protein D (PD)	Glycerophos-phodiesterase, binds IgD	AlPO_4_ + MPL, Freund’s adjuvant, OMVs	Animal studies, licensed as antigenic carrier protein in PHiD-CV pneumococcal conjugate	Kennedy et al. (2000), Forsgren et al. (2008), and Davoudi Vijeh Motlagh et al. (2016)
PD + PE-PilA	A trivalent vaccine containing10 μg PD and 10 μg PE-PilA fusion	Alum, AS01_E_	Phase 1 and 2 completed	Leroux-Roels et al. (2016) and Wilkinson et al. (2019)
PD + PE-PilA+UspA2	A multicomponent vaccine containing antigens of three surface proteins from NTHi and one from Mcat	AS01_E_	Phase 1 and 2 completed	Van Damme et al. (2019), De Smedt et al. (2021), and Galgani et al. (2022)
Hap	Adhesin, IgA protease-like autotransporter protein	None or mutant cholera toxin (CT-E29H)	Animal studies. Immunization with Hap protected against intranasal challenge of NTHi in mice.	Cutter et al. (2002) and Bafroee et al. (2016)
Hia	Adhesin, Hsf in type b strains.	Freund’s adjuvant	Animal studies. Immunization with recombinant adenovirus vaccines expressing the Hia protected against NTHi OM in chinchillas.	Winter and Barenkamp (2009), and Winter and Barenkamp (2014)
HMW1, HMW2	Adhesins	Freund’s adjuvant	Animal studies and *in vitro* human specimen tests. Immunization with HMW1/HMW2 mixture partially protected against NTHi OM in chinchillas.	Winter and Barenkamp (2003, 2014)
Protein E (PE)	Adhesin	Alum	Animal studies. Induced protection in a mouse pulmonary challenge model with NTHi	Ronander et al. (2009) and Behrouzi et al. (2017)
Protein F (PF)	Adhesin, ABC transporter	Freund's adjuvant, Alum	Animal models and *in vitro* human specimen tests. Immunization with protein F protected against the intranasal challenge of an NTHi strain in mice.	Jalalvand et al. (2014)
PilA2	Type IV Pilus, involved in adherence, twitching motility and biofilm formation	dmLT (A double mutant form of *E. coli* heat-labile enterotoxin)	Animal studies. The recombinant soluble form of PilA inhibited NTHi biofilm formation *in vitro* and hindered onset of NTHi-induced OM in a chinchilla model.	Novotny et al. (2015a) and Mokrzan et al. (2016, 2018)
PE-PilA fusion	A fusion protein containing PE and PilA sequence	Alum, ASO1, ASO4	Animal studies. PE-PilA immunized mice showed significant protection against intranasal NTHi challenge. Passive transfer of antiserum to Protein E-PilA prevented NTHi-induced OM in chinchillas.	Ysebaert et al. (2019)
P5 fimbrin	Adhesin, binds mucin, OMP A like protein	Freund's adjuvant	Animal studies and in vitro human specimen tests. Immunization with P5 enhanced lung clearance and ME clearance of NTHi in rats. Immunization with P5 enhanced ME and NP clearance of NTHi in chinchillas.	Bakaletz et al. (1999), Novotny et al. (2002), and Kyd et al. (2003)
ChimV4	A fusion protein containing P5 and rsPilA	dmLT	Animal studies	Novotny et al. (2015a, 2017) and Novotny and Bakaletz (2020)
P6	Peptidoglycan-associated lipoprotein involved in immunomodulation and induction of bactericidal antibody responses	Freund’s adjuvant, alum, CT, *α*-galactosylceramide, FMS-like tyrosine kinase receptor 3 ligand as a mucosal adjuvant	Animal studies and *in vitro* human specimen tests. Immunization with P6 protected against NTHi OM in chinchillas.	DeMaria et al. (1996), Bakaletz et al. (1999), and Kodama et al. (2000, 2011b)
P6 + CCL20	A nasal NTHi vaccine containing P6 and the chemokine CCL20	None	Animal studies	Kodama et al. (2011a, 2011b)
cCHP-P6 nanogel vaccine	A cationic cholesteryl pullulan-based nasal vaccine containing P6 protein	None	Animal studies	Nakahashi-Ouchida et al. (2022)
OMP 26	Translocation of OMPs and LOS	Freund’s adjuvant, Ribi adjuvant, S5 (aluminium salts, monophosphoryl lipid A, and QS21)	Animal studies and *in vitro* human specimen tests. Immunization with OMP26 enhanced lung clearance of NTHi in rats. Immunization with OMP26 induced clearance of NTHi from the chinchilla ME and NP.	Kyd and Cripps (1998), Kyd et al. (2003), and Pichichero et al. (2010)
Lipidated OMP26 & P6	Lipidated recombinant P6 and OMP26	Aluminium hydroxide, Curdlan	Animal studies. Lipidation of antigens enhanced immunogenicity by stimulation of TLR2 receptors and Th17 cells and, consequently improved protection against NP colonization and ME infections caused by NTHi in mice	Kaur and Pichichero (2022)
EF-Tu	Intracellular elongation factor thermal-unstable, surface protein	Freund’s adjuvant	Animal studies and *in vitro* human specimen tests.	Thofte et al. (2018, 2019)
Surface-exposed proteins (SEPs)	A vaccine containing synthetic peptides corresponding to some highly conserved surface-exposed regions	Freund’s Adjuvant	Animal studies	Whitby et al. (2015)
Hi Poly 1	A bacterial vaccine polypeptide, comprising 9 unique peptides from 6 different surface proteins of NTHi	NA	Animal studies. Hi Poly 1-immunized chinchillas cleared NTHi infection faster than the control group.	Whitby et al. (2020)
KDO	Ketodeoxyoctanoate, a sugar unique to Gram-negative bacteria which is used to decorate LOS during active infection	NA	*In vitro* assays. Monoclonal antibody raised against an NTHi KDO was shown to be bactericidal against 12 out of 33 NTHi strains tested.	Apicella et al. (2018)
OMVs	Outer membrane vesicles	Freund’s Adjuvant, MPLA-CpG adjuvant	Animal studies. OMV-immunized animals were completely protected against OM and OMV-specific antisera had opsonophagocytic activity against many HMW1/HMW2-expressing NTHi strains.	Winter and Barenkamp (2017) and Behrouzi et al. (2020)

ABC, adenosine triphosphate-binding cassette; AS01, adjuvant system 01; AS04, adjuvant system 04; AS0_E,_ adjuvant system 0E; CT, cholera toxoid; cCHP, cationic cholesteryl pullulan; CCL20, Chemokine ligand 20; CpG, Unmethylated cytosine–guanine dinucleotide; dmLT, double mutant heat-labile toxin of *E. coli*; FMS, feline mcDonough sarcoma; Hap, haemophilus adhesin protein; Hia, *Haemophilus influenzae* adhesin; HMW, high molecular weight protein; Hi Poly 1, *Haemophilus influenzae* polypeptide 1; IgD, Immunoglobulin D; KDO, ketodeoxyoctonic acid; LOS, lipo-oligosaccharide; MPL/MPLA, monophosphoryl lipid A; *Moraxella catarrhalis*, *Moraxella catarrhalis*; ME, middle-ear; NA, not available; NP, nasopharynx; NTHi, non-typeable *Haemophilus influenzae*; OM, otitis media; OMP, outer membrane protein; OMVs, outer membrane vesicles, P5, outer membrane protein 5; P6, outer membrane protein 6; PilA, pilus A; PhtD, pneumococcal histidine triad D; QS-21, *Quillaja saponaria* derived saponin 21; SEPs, Surface-exposed proteins; Th17, T helper 17 cells; TLR2, toll-like receptor 2.

#### 2.2.5 Recombinant polypeptides as vaccine candidates for NTHi, and use of reverse vaccinology

As noted above, combining protein antigens can be achieved by identifying immunologically relevant epitopes, and generating fusion proteins (see PE-PilA and ChimV4). Since bacterial genomes have been available, bioinformatics analysis has identified putative surface-exposed proteins, in an approach described as “reverse vaccinology,” an approach that contributed to antigens in the GlaxoSmithKline vaccine against serogroup B meningococcus (Masignani et al., 2019). Significant improvements in bioinformatic analysis tools, availability of increasing numbers of genome sequences for each bacterial species, and increased understanding of immunologically relevant sequences and structure allow more sophisticated and rapid screening for candidate proteins, domains, or short peptide sequences. Recently Whitby et al. identified 56 putative surface proteins conserved in 26 genomes (selected to represent diverse strains). Potential surface exposed regions were identified using molecular modeling. Antisera were raised in rats against 10 synthetic peptides corresponding to surface-exposed regions highly conserved in strains. Five of the antisera were protective in the infant rat model of invasive NTHi infection establishing their *in vivo* efficiency (Whitby et al., 2015). A different bioinformatics platform identified two similar proteins as being future NTHi vaccine candidates (D’Mello et al., 2019).

The same group extended their strategy to produce a single polypeptide comprising 9 unique peptides from 6 different surface proteins. Immunization of chinchillas with this polypeptide resulted in faster clearance of NTHi-induced OM (Whitby et al., 2020). This approach has the potential to be applied to other pathogens that have complex diversity in surface antigens, including Mcat. In the coming years, it can be anticipated that high throughput bacterial genomics, computational-based vaccine candidate identification approaches, and experimental validation will identify additional vaccine candidates for NTHi.

### 2.3 Mcat vaccines

Mcat is a Gram-negative diplococcus that is typically present in the NP of most infants and children. Mcat is commonly associated with OM, however, the exact percentage of Mcat-induced OM differs between studies because of alterations in sample collection, methods of bacterial detection, and geographical location. It is the first otopathogen that colonizes the NP and the first otopathogen to initiate an episode of OM (Kaur et al., 2014a; Ngo et al., 2016), in some susceptible populations colonizing within a month of birth (Beissbarth et al., 2021). Mcat, like NTHi, is also a key pathogen in exacerbations of COPD (Murphy et al., 2019). Mcat can be treated with antibiotics, but almost all strains are now beta-lactamase positive, potentially promoting the survival of otherwise beta-lactam-sensitive bystander bacteria (Verduin et al., 2002).

Mcat vaccine can help prevent NTHi and pneumococcal infections by excluding a potential co-pathogen that can induce passive protection from β-lactam antibiotic therapy (Perez and Murphy, 2017). However, Mcat vaccine development is lagging behind that of *S. pneumoniae* and NTHi. Learning from the NTHi vaccine approach, it is likely that an effective Mcat vaccine may need multiple antigens, as it also has a range of antigenically and phase variable antigens. Nevertheless, various factors deter the progress of Mcat vaccine development, including the absence of a known correlate of protection and a suitable animal model mimicking Mcat infection in humans (Perez and Murphy, 2019) (and see section below on animal models). Regardless of these barriers, noteworthy strides have been made in the last few years in the identification of potential antigens, efficient adjuvant formulations, and immunization routes using both computational and experimental methods [see Table 3 and a review by (Perez and Murphy, 2017)]. However, several potential vaccine candidates showed initial promise, but for which there are few recent reports of progress. This may reflect the lower priority that Mcat appears to have or the lack of high-throughput animal models of OM that can be used to test their efficacy.

**Table 3:** Potential vaccine antigens of *Moraxella catarrhalis* at various stages of development.

Antigen	Putative antigen function/other features	Adjuvant	Current development stage	References
UspA2	Adhesin and autotransporter; involved in serum resistance and other virulence mechanisms	QS-21, Conjugated to *d*LOS + Ribi-700	Phase 2 completed	Tan et al. (2006), Ysebaert et al. (2021), Andreas et al. (2022), and Arora et al. (2022)
OMP CD	Adhesin, binds mucin	Alum, MPL + Alum, QS-21, conjugated to *d*LOS + Ribi-700, IFA, AdDP	Animal studies. Enhanced lung clearance of bacteria in immunized mice (MPCM).	Murphy et al. (1999, 2005) and Liu et al. (2007)
Hag/MID	Functions as an adhesin and hemagglutinin, binds IgD	CFA/IFA	Animal studies. Enhanced lung clearance of bacteria in immunized mice (MPCM).	Forsgren et al. (2004), Bullard et al. (2005), and LaFontaine et al. (2009)
MhaB1, MhaB2	Adhesin	CFA/IFA	Animal studies. Enhanced lung clearance of bacteria in immunized chinchilla (CNCM).	Balder et al. (2011) and Shaffer et al. (2013)
CysP	The substrate binding protein of ABC transport system, binds sulfate	IFA	Animal studies. Enhanced lung clearance of bacteria in immunized mice (MPCM).	Murphy et al. (2016)
AfeA	Substrate binding protein of an ATP binding cassette (ABC) transporter, binds ferric ions	CFA/IFA	Animal studies. Induced protective responses in the mouse pulmonary clearance model following challenge with Mcat.	Murphy et al. (2017)
OppA	SBP of ABC transporter, binds peptides	CT	Animal studies. Enhanced lung clearance of bacteria in immunized mice (MPCM).	Yang et al. (2011) and Ren et al. (2019)
SBP2	Substrate binding protein of an ABC transporter, binds arginine	IFA	Animal studies. Enhanced lung clearance of bacteria in immunized mice (MPCM).	Otsuka et al. (2014, 2016)
Msp22	Surface lipoprotein, binds heme	IC3, IFA	Animal studies. Enhanced lung clearance of bacteria in immunized mice (MPCM).	Ruckdeschel et al. (2008) and Smidt et al. (2013)
Msp75	Homology to succinic dehydrogenase	CT	Animal studies. Enhanced lung clearance of bacteria in immunized mice (MPCM).	Ruckdeschel et al. (2008, 2009)
M35	Porin, involved in the uptake of energy resources	None	Animal studies. Enhanced lung clearance of bacteria in immunized mice (MPCM).	Easton et al. (2005, 2008, 2011)
dLOS	Adhesin, endotoxin	Conjugated TT or HMP + Ribi-700, Conjugated CRM9 + CT, Conjugated CRM9 + Ribi-700	Animal studies. Enhanced lung clearance of bacteria in immunized mice (MPCM).	Jiao et al. (2002), Yu and Gu, (2005, 2007), and Ren et al. (2011a)
BamA	Outer membrane protein assembly factor	NA	Predicted through *in silico* analysis	Soltan et al. (2021)
LptD	LPS assembly protein	NA	Predicted through *in silico* analysis	Soltan et al. (2021)
OMP G1a, OMP G1b	Lipoprotein, putative copper transport protein	NA	NA	Adlowitz et al. (2004, 2006)
PilA	The major protein subunit of TFP; involved in natural genetic transformation, biofilm formation, and adherence	NA	NA	Ren et al. (2019)
McaP	Adhesin, autotransporter	NA	NA	Timpe et al. (2003) and Lipski et al. (2007)
OMP E	Fatty acid transport	NA	NA	Bhushan et al. (1997)

ABC, ATP-binding cassette; AdDP, adamantylamide dipeptide; AfeA, substrate binding protein of an ATP binding cassette (ABC) transporter; BamA, β-barrel assembly machinery; CNCM, chinchilla nasopharyngeal colonization model; CysP, sulphate binding protein of *Moraxella catarrhalis*; CFA, complete Freund’s adjuvant; CRM, Cross-reactive mutant of diphtheria toxoid; CT, cholera toxoid; dLOS, detoxified lipo-oligosaccharide, Hag, hemagglutinin; HMP, high molecular weight proteins; IFA, incomplete Freund’s adjuvant; IgD, Immunoglobulin D; IC31, a novel two-component adjuvant; LptD, LPS transport protein D; LbpB, lactoferrin-binding proteins; mAb, monoclonal antibodies; MPL, monophosphoryl lipid A; MPCM, mouse pulmonary clearance model; MID, Moraxella immunoglobulin D-binding protein; M35, Moraxella porin 35; Mcap, *Moraxella catarrhalis*-adherence protein; Mha, *Moraxella catarrhalis* filamentous hemagglutinin adhesin-like protein; Msp., Moraxella surface protein; NA, not available; OMP, outer membrane protein; OppA, oligopeptide permease protein A; PilA, pilus A; QS-21, *Quillaja saponaria* derived saponin 21; SBP, substrate-binding protein; TbpB, transferrin binding protein B; TFP, type IV pili; TT, tetanus toxoid; UspA2, ubiquitous surface protein A2.

#### 2.3.1 Adhesin proteins as vaccine candidates

As discussed earlier, presently only one Mcat protein antigen has been tested in clinical trials (UspA2). UspA2 interacts with host structures and extracellular matrix proteins and induces bacterial adherence and serum resistance (Singh et al., 2010; Su et al., 2013; Singh et al., 2015). Vaccination with UspA2 induced protective antibodies in mice (Chen et al., 1996). UspA2 has been reported to have variations in sequence and structure (Su et al., 2013), which may affect antibody recognition and binding. Despite this, anti-UspA2 antibodies raised in mice, rabbits, and guinea pigs were able to induce complement-mediated killing of many, but not all Mcat strains of different origins (Ysebaert et al., 2021). The NTHi-Mcat vaccine containing UspA2 was immunogenic in the phase 1 clinical trial, with a satisfactory safety profile both in healthy individuals and adults with a smoking history (Van Damme et al., 2019). A 4-year follow-up (NCT03201211) found that immune responses to NTHi antigens persisted over an extended period, whereas UspA2 did not (De Smedt et al., 2021). However, in all human trials to date, baseline titers of serum antibodies against UspA2 are relatively high, suggesting either natural immune responses to UspA2 or similar proteins in Mcat, or cross-reactivity with an antigen from another source. This observation complicates the interpretation of data in clinical trials on the persistence of serum antibody responses. In a randomized, placebo-controlled, phase 2b trial (NCT03281876) of adults with a history of acute exacerbations of COPD, this vaccine showed good safety, however, did not demonstrate efficacy in lowering the yearly rate of severe or moderate exacerbations of COPD (Andreas et al., 2022; Arora et al., 2022).

#### 2.3.2 Mcat vaccine antigens validated by reports of human natural responses

Several antigens have shown promise in early animal trials but have few recent reports. This includes Hemagglutinin Moraxella IgD binding protein (Hag/MID), PilA2 (the major protein subunit of Tfp), and outer-membrane protein CD (OMP CD). However, they have been identified as targets of natural antibody production in COPD patients, or children (LaFontaine et al., 2009; Ren et al., 2019). OMP CD is an adhesin and a porin protein (Murphy et al., 2003) with surface-exposed epitopes and is considered conserved among different Mcat strains (Sarwar et al., 1992; Murphy et al., 1993). Murine immunization with OMP CD increased Mcat clearance following pulmonary challenge (Liu et al., 2007). Hag/MID is an OMP involved in human erythrocyte agglutination (Pearson et al., 2002; Forsgren et al., 2003; LaFontaine et al., 2009). Hag/MID shows sequence diversity among different strains, however, the adhesive domain region is conserved. A truncated Hag/MID induced a protective response in mice (Forsgren et al., 2004). PilA2 was present in 57.5% of 106 tested clinical Mcat isolates (Luke-Marshall et al., 2011). It was required for the adherence of Mcat to *in vitro* cultured human epithelial cells and NP colonization in a chinchilla model (Luke et al., 2004, 2007). At present, no data is available on measuring the immune responses to the PilA2-based vaccine in animal models or humans. Ren et al. studied natural antibody production against OMP CD, Hag, and PilA2 in stringently defined otitis-prone (sOP) children compared to non-otitis-prone children (NOP). All proteins elicited antibodies, but the sOP population showed reduced serum antibody responses (Ren et al., 2019). Serum and sputum sample analysis from COPD patients revealed that a Hag/MID domain encompassing amino acids 706 to 863 was a target of serum IgG and sputum IgA (LaFontaine et al., 2009).

#### 2.3.3 Mcat vaccine antigens with animal data but no recent reports

*Moraxella catarrhalis*-adherence protein (McaP) is an adhesin and an autotransporter having esterase and phospholipase B activities (Timpe et al., 2003). Moreover, this protein is highly conserved as 98–100% amino acid sequence similarity was observed among 8 studied strains (Timpe et al., 2003; Lipski et al., 2007). Mouse anti-Mcap serum hindered the binding of Mcat and recombinant *E. coli* expressing McaP to human respiratory epithelial cells (Timpe et al., 2003; Lipski et al., 2007). Outer-membrane protein E (OMP E) is a porin and is involved in the binding and transport of fatty acids. Gene sequence analysis showed that the *ompE* gene stayed stable during the colonization process (Martin et al., 1993). OMP E also expressed a highly conserved surface epitope (Bhushan et al., 1997). Further investigations are needed to gain a clear understanding of McaP and OMP E immunogenicity, its different antigenic domains, and the protective immune responses they elicit. M35 is an OMP that acts as a general porin and is essential for energy source uptake for Mcat (Easton et al., 2005). M35 is suggested to be essential for *in vivo* colonization and resistance mechanisms (Easton et al., 2008). M35 gene sequence showed high conservation (99.6–100%) among 18 tested isolates. In immunoblot analysis, mouse anti-M35 serum displayed binding to whole-cell protein preparations from all the tested isolates (Easton et al., 2005). Anti-sera from M35 immunized mice did not show bactericidal activity, however, it improved opsonic activity. In another study, mucosal immunization of mice with recombinant M35 through intra Peyer’s patch increased clearance of bacteria from the lungs of Mcat-challenged mice (Easton et al., 2011).

*Moraxella catarrhalis* filamentous hemagglutinin adhesin-like protein (Mha)B1 and MhaB2 (exoproteins), and MhaC (transporter) (Balder et al., 2007), which are also named *M. catarrhalis* hemagglutinin-like protein (Mch)A1 and MchA2 for the secreted proteins and MchB for the transporter are potential vaccine candidates (Plamondon et al., 2007). A mutant Mcat lacking MhaB1 and MhaB2 could not colonize the chinchilla NP, emphasizing the importance of these proteins. Immunization of chinchillas with a polypeptide shared by MhaB1 and MhaB2 induced antibodies that interfered with the colonization of Mcat and promoted bacterial clearance (Balder et al., 2011; Shaffer et al., 2013).

#### 2.3.4 Proteins involved in nutrient acquisition as potential antigens

Murphy et al. screened the genome of Mcat strain ATCC 43617 for potential surface proteins. This identified several substrate-binding proteins (SBPs) of the ABC transporter family. The group evaluated CysP, AfeA, OppA, and Sbp2 for their immunogenic potential which are studied by other groups as well (Yang et al., 2011; Otsuka et al., 2014; Murphy et al., 2016; Otsuka et al., 2016; Murphy et al., 2017). CysP is involved in the uptake of sulfate, AfeA binds ferrous, ferric, zinc, and manganese ions, OppA is likely an oligopeptide binding protein of the oligopeptide permease ABC transport system, and Sbp2 mediates the uptake of arginine, a strict growth requirement of Mcat. All were found to express accessible surface epitopes, to be conserved within Mcat strains, and to provide enhanced clearance in a lung challenge model in mice (Yang et al., 2011; Otsuka et al., 2014; Murphy et al., 2016, 2017).

#### 2.3.5 Other OMPs as vaccine candidates

Moraxella surface proteins (Msp) including Msp22, Msp75, and Msp78 showed 97–99% homology in amino acid sequence among 10 tested strains (Ruckdeschel et al., 2008). Mucosal and systemic immunizations of mice with recombinant Msp22 and Msp75 induced IgG and IgA antibodies. Mouse and rabbit anti-sera to recombinant Msp22 and Msp75 were able to recognize corresponding proteins in numerous Mcat strains. In addition, intranasal immunization of mice with recombinant Msp22 substantially lowered the bacterial load in the lungs of Mcat challenged mice (Ruckdeschel et al., 2009; Smidt et al., 2013). Therefore, these proteins are attractive Mcat vaccine antigens, nonetheless, more studies are required to uncover their detailed functions and immunogenicity in humans.

#### 2.3.6 Peptide-based vaccines against Mcat

Peptide-based vaccines offer numerous advantages, such as the exclusion of deleterious parts from full-length antigens, ease of chemical modification, absence of infectious material, and ease of production and storage. Although they generally have poor immunogenicity, this can however be compensated for by using modified formulations of the vaccine (Purcell et al., 2007). Lactoferrin-binding protein A (LbpA) is a receptor for human lactoferrin and is involved in iron transport. Whole LbpA, both native and recombinant was reported to be non-immunogenic (Du et al., 1998; Yu et al., 1999). Yassin et al. used immune-informatics analysis to identify a peptide from LbpA named peptide A which showed high immunogenicity in mice and successfully cleared Mcat from mouse lungs. Furthermore, anti-peptide LbpA antibodies were bactericidal against heterogeneous Mcat strains (Yassin et al., 2016). Peptide A is the first promising peptide-based vaccine against Mcat, and it warrants further investigation.

#### 2.3.7 Mcat LOS as a vaccine candidate

LOS comprises a major virulence factor of Mcat and performs a vital role in eliciting inflammatory immune responses (Hassan et al., 2012). It also mediates serum resistance (Zaleski et al., 2000) and adherence to human epithelial cells (Peng et al., 2005). LOS from Mcat is relatively conserved, with three major serotypes (Holme et al., 1999; Verduin et al., 2002; Ren et al., 2011b), and no phase variation within a strain (unlike NTHi). In common with CPS, LOS glycans are poorly immunogenic unless conjugated to a carrier protein. Removal of toxic Lipid A moieties is also necessary. Detoxified LOS (*d*LOS) is conjugated to a carrier protein. Immunization of rabbits and mice with the conjugates derived from all three serotypes induces significant levels of antigens-specific mucosal and serum antibodies. Bactericidal antibodies were also identified in immunized animals (Jiao et al., 2002; Yu and Gu, 2005, 2007). Ren et al. generated two mutant LOS from Mcat strain O35E to produce conserved LOS antigens and exclude a potential autoimmune response in humans. These mutant LOS had one or two terminal galactopyranose (Gal*p*) residues deleted and conjugated to tetanus toxoid (TT). These conjugates exhibited broad-spectrum protection and induced high levels of serum IgG with bactericidal activity against all three serotypes (Ren et al., 2011b). In another study, *d*LOS-protein conjugates from all three serotypes were combined and immunized mice developed humoral and cell-mediated immunity which enhanced pulmonary clearance of six strains of all three serotypes of Mcat (Ren et al., 2011a). In a similar approach, truncated (common) LOS was conjugated to recombinant OMP26 of NTHi. This elicited complement-mediated bactericidal activity against tested strains of serotype A and Mcat and one NTHi strain (Singh et al., 2020).

#### 2.3.8 Mcat vaccine targets identified by reverse vaccinology: promising future candidates

As mentioned above, D’Mello et al. used a computational approach to identify potential candidate antigens against a range of criteria, from NTHi and Mcat (D’Mello et al., 2019). Top Mcat candidates identified included a porin protein, an iron transporter protein, and 2 unstudied conserved-hypothetical proteins (D’Mello et al., 2019). Soltan et al. applied a different reverse vaccinology approach to identify potential protein candidates, including criteria of being essential, outer membrane-localized, involved in virulence, antigenic with no human homologs, with appropriate molecular weight and less than two transmembrane helices. Only LPS assembly protein LptD and outer membrane protein assembly factor BamA met these criteria. From this, several peptides of each were combined to construct a theoretical multi-epitope candidate peptide (Soltan et al., 2021). It is likely that future publications will reveal which of these are validated and can be further developed, but as with NTHi, a multi-component vaccine is likely required.

In summary, numerous Mcat antigens have revealed outstanding immunogenicity, induced functional antibodies, and generated protective responses in animal models. Nonetheless, there has been no clinical testing on them. Future studies are needed on these antigens to make advances in Mcat vaccine development.

## 3 OM animal models

Animal models are an important tool to study, (1) pathogens of OM; (2) the microenvironment during disease progression; (3) how pathogen-specific immunity helps resolve acute OM; and (4) vaccination strategies against this disease before they can be applied to humans. Over the years, several animal models have been developed for studying OM (see a recent review by (Davidoss et al., 2018)). There was much interest in the use of guinea pigs as an animal model in the 1960s and 1970s, mainly because of their popularity in other branches of medical research. However, since the late 1970s, the chinchilla has been used for investigating OM. This is primarily due to the chinchilla’s large bulla that makes access to the ME easy. In the 1980s, gerbils were popular because of their propensity to develop cholesteatoma (Bakaletz, 2009). However, since 2010 interest in mouse models of OM has increased significantly because of the increased ease of genetic manipulation, and identification of mouse host mutants with susceptibility to OM (Geng et al., 2019). A summary of these animal models is provided in Table 4. Since most human bacterial pathogens do not naturally colonize or cause disease in animals, infection of ME or NP in animals can yield different immune responses to that in humans (Sabirov and Metzger, 2008). For a given model, a pathogenic organism must meet certain criteria to be considered pathogenic that is: (a) the pathologies induced by the pathogen in the animal must resemble those observed in humans; (b) otomicroscopy, tympanometry, and histopathology, pathologies can be objectively recorded; and, (c) the organism is able to reproduce in the ME space (Doyle, 1989).

**Table 4:** A comparison of different animal species in OM models.

Animal	Strengths	Limitations	References
Rat	(i) ME anatomy, histology, and ET opening pressure have a resemblance to that of humans (ii) Medium-sized bulla (iii) Does not develop sepsis easily (iv) Well-defined pharmacokinetic parameters and gene sequences	(i) Not easily manipulated (ii) Fragile junction of the tympanic bulla (iii) Can develop spontaneous AOM (iv) Costly	Hardy et al. (2001), Cayé-Thomasen and Tos (2002), Piltcher et al. (2002), and Chaney et al. (2011)
Mouse	(i) Mouse genome and the immune system are extensively described (ii) Low cost (iii) Small size makes housing comparatively easy/cost efficient (iv) Numerous transgenic and knockout species are available (v) Wide availability of experimental reagents	(i) Relationship between age and resistance of the tympanic membrane which may lead to alteration of the ME response (ii) ME and TM are ‘small’ in size - difficult to perform surgical procedures (iii) Susceptible to anaesthetic drugs (iv) Large and patent ET (v) Thin tympanic membrane	Ryan et al. (2006), Zheng et al. (2006), and Tyrer et al. (2013)
Chinchilla	(i) Easy access to ME for surgery - large bulla (ii) Rarely develop spontaneous AOM (iii) Ear has similar structures to the human ear (stapes, cochlea, distribution of hair cells, and vestibular system) (iv) Susceptible to human ME pathogens	(i) Relatively high purchase cost (ii) Not readily available in many countries (iii) Easily develops general sepsis with a high mortality rate upon infection (iv) Difficult to access the auditory canal	Bakaletz (2009), Shaffer et al. (2013), and Wolter et al. (2014)
Mongolian gerbil	(i) Low cost (ii) Small size (iii) Relatively large ME (iv) Rarely develop natural OM however susceptible under laboratory conditions	(i) Small external auditory canal	Chole et al. (1981), Unge et al. (2009), and Yamamoto-Fukuda et al. (2011)
Guinea pig	(i) Low cost (ii) Easily handled in surgical experiments (iii) Straightforward ME inoculation procedure (iv) The anatomy of the temporal bone, the cochlea and its components, and the vestibulocochlear nerve resembles the humans	(i) Small external auditory canal and ME (ii) Difficult to induce OM (iii) Differences in ME anatomy, immune response, and pharmacokinetic profiles of medications	Dai et al. (2009) and Guan and Gan (2013)
Monkey	(i) Recommended in the analysis of cerebral cortex function in central processing deficits, because this area finds more similarities between monkeys and humans than between humans and rodents (ii) There are similarities between monkeys and humans in progressive hearing damage, which increases in severity with aging	(i) Costly and less available (ii) Can be difficult to handle in the laboratory, because they are aggressive and susceptible to diseases (iii) The ET is shorter and more flexible, especially in the first years of life, and the physiological function is lower due to the paratubal muscles’ anatomy (iv) Use is associated with a higher negative psychosocial effect	Dohar et al. (1998, 2005), Lavinsky and Goycoolea (1997), Kaas and Hackett (2000), and Engle et al. (2013)

AOM, acute otitis media; ET, Eustachian tube; ME, middle ear; OM, otitis media.

### 3.1 Routes of pathogen administration

To mimic human OM, an ideal model is one in which intranasal (IN) instillation of bacteria leads to NP colonization, and ascent of bacteria through the ET to the ME. As host-adapted pathogens, the three major otopathogens exhibit the differential capacity to survive, colonize, replicate, and directly ascend to the ME. Some animal models of OM use direct instillation of bacteria into the ME.

In the IN-inoculation method, the entry portal for the pathogen into the ME is analogous to the human disease process, because NP is the first place where bacteria and viruses colonize and reproduce before invading the ME cavity. This method can be highly reproducible if inoculum volume is maintained with negligible aspiration or swallowing of the inoculum which can be assisted through aesthesia before IN inoculation. Commonly used microorganisms to induce OM in experimental settings are *S. pneumoniae*, NTHi, methicillin-resistant *S. aureus,* and *Pseudomonas aeruginosa*. Some animal models use prior infection, or co-infection of the virus to facilitate bacterial OM (Appell et al., 1971; Meek III et al., 1999; McCullers and Rehg, 2002; Faisca and Desmecht, 2007; McCullers et al., 2007). Notably, Mcat will rarely survive in the murine mucosa. Bacteria can be inoculated directly into the bullae, or by trans-tympanic inoculation into the ME, leading to the induction of OM. These methods have high reproducibility and precision as the exact number of microorganisms in the inoculum can be determined leading to less variability between individual animals. A drawback of these methods is the bypass of NP colonization. Furthermore, surgical skills are required to use the trans-bullar approach. Inconsistency in this process can cause damage to the nearby blood vessels (Parks et al., 1996; Pinilla et al., 2001). In comparison, the trans-tympanic approach is easier to perform, using a fine needle to deliver the inoculum through the tympanic membrane. Sometimes, injected material can drain through the tympanic membrane hole leading to imprecise inoculum volume as compared to intra-bullar inoculation. The hole made in the tympanic membrane can be a channel for contamination. In addition, pressure equilibration can result in increased drainage of ME effusions through this hole. Thus, the trans-tympanic delivery method is appropriate for those species having direct trans-canal access (Ryan et al., 2006).

Non-infectious animal models have also been reported. Several mouse models have been generated by utilizing LPS or peptidoglycan-polysaccharides (Li et al., 2015; Zhang et al., 2015; Kim et al., 2016). Ovalbumin has been used to induce an eosinophilic OM via IN administration in a mouse model or a trans-tympanic injection in a guinea pig model (Matsubara et al., 2014; Kim et al., 2016). Moreover, histamine has been used in guinea pigs to induce AOM (Kozan et al., 2015).

OM induction strategies are therefore dependent upon the experimental requirements and the type of animal being used. The IN-challenge model is preferred for inducing OM while assessing protective immunity in ME against NP colonization. A direct ME challenge could, however, be preferred for determining the extent of ME inflammatory responses as well as studying the protective immunity against ME challenge.

#### 3.1.1 OME induction

Several animal models have been developed that examine the pathological changes associated with OM, in the absence of pathogen challenge. The principal anatomical cause of OME is ET dysfunction. To mimic this, animal models of OME are generated through ligation or cauterization of ET utilizing a trans-oral or trans-neck approach (Piltcher et al., 2002), or by injecting chemical materials through the tympanic membrane (Aynali et al., 2011). For the first approach, the ET orifice is cauterized, and the cartilage portion of the ET is ligated with nylon or an electrical cautery (Piltcher et al., 2002). Cauterization or ligation of the ET is however an irreversible process. The trans-neck method is a precise approach for blocking the tube, but it is more laborious as compared to the trans-oral approach. On the other hand, the trans-oral approach is easy, however, it is difficult to get consistent results with this method (Huang et al., 2012). Injecting chemicals via the tympanic membrane is comparatively simpler to perform than the methods mentioned above. Injection of histamine solution has been used to induce OME in rats (Aynali et al., 2011).

### 3.2 Animal models

The choice of the experimental animal depends on anatomical, physiological, economic, spatial, and psychological factors, as well as experimental objectives. Depending on the research objective, animal model chosen, and access to equipment, there may be variations in studies. Therefore, it is crucial to understand the characteristics of the chosen model’s auditory system, along with its advantages, disadvantages, and limitations.

#### 3.2.1 Rats

Rats exhibit the highest compatibility in terms of ME anatomy and histology to human infants and children as compared to other rodents, therefore, they are preferred models of AOM (Cayé-Thomasen and Tos, 2002). In addition, most human pathogens readily infect rat ME (Chaney et al., 2011) and the opening pressure of the ET is equivalent to that in humans (Hellstorm and Stenfors, 1983). The rat AOM course bears a close resemblance to that of humans (Hermansson et al., 1988), and ciliary clearance tracts and histological cell types are also analogous to humans (Daniel III et al., 1982). As a result of the relatively large tympanic bulla in the rat, bacteria can easily be inoculated into ME via the tympanic membrane or the bulla. Once infection has occurred, it usually resolves within 10–12 days without ME effusion signs and affecting tympanic membrane preservation (Hermansson et al., 1988; Prellner et al., 1999). It is also possible to induce a persistent OME in rats which can last for more than 16 weeks upon using appropriate inoculum (Piltcher et al., 2002). Moreover, rats are not prone to develop general sepsis which further enhances their usefulness (Prellner et al., 1999). Rat models of AOM with *S. pneumoniae* and OME model with ET obstruction by dental material are reported (Hermansson et al., 1988; Piltcher et al., 2002). An allergen-induced OME rat model was established using an intraperitoneal injection of ovalbumin and a subsequent intratympanic injection of ovalbumin into the ME (Hardy et al., 2001; Zhang et al., 2022). Rat OM model with methicillin-resistant *S. aureus* and *P. aeruginosa* injected into the ME cavity via the tympanic membrane have been developed (Yadav et al., 2017). Magnuson et al. utilized *Haemophilus influenzae* type b and NTHi strain 3,655 to inoculate the ME of Sprague–Dawley rats and studied the course of AOM development. Rats developed AOM with both bacterial strains 4 days post-inoculation (Magnuson et al., 1997). Rat models of OM with cholesteatoma have also been developed utilizing different chemical compounds such as dimethyl-benzanthrancene (DMBA) or propylene glycol injections into the ME (Klein-Szanto et al., 1982; Huang et al., 1988). An acute secretory OM (SOM) rat model was developed by delivering endotoxin into the ME cavity through the eardrum (Li et al., 2021). Mcat can induce OM in rats, but only with high dose, trans-tympanic inoculation, and OM is shorter lived than that caused by NTHi or *S. pneumoniae* (Westman et al., 1999).

#### 3.2.2 Chinchilla

Another rodent that is favored for AOM research is the chinchilla which displays general susceptibility to human bacterial and viral pathogens of OM (See review by (Bakaletz, 2009)). An advantage of using the chinchilla is that its tympanic membrane is almost the same size as humans. In addition, its large bulla facilitates the inoculation of pathogens and collection of ME effusions (Watanabe et al., 1982). The temporal progression and natural history of the disease process resemble human OM (DeMaria et al., 1996; Bakaletz, 2009). However, this species has a multi-loculated bulla, which is easily prone to fibrosis, with the ability to seal a chamber of the bulla and prevent infection from spreading. The external auditory canal of chinchillas is elongated and S-shaped, making it difficult to inspect the tympanic membrane with an ordinary otoscope and making the trans-tympanic challenge more difficult. It is only widely available in North and South America; therefore, lack of easy availability hinders its application. The rate of inner ear complications is high in chinchillas, and they tend to develop general sepsis, especially the younger chinchillas, and have a high mortality rate. Furthermore, chinchillas are not as capable of withstanding adverse conditions in laboratory settings as rats. Chinchillas tend to shed their fur at the insignificant sign of distress (Murrah et al., 2015).

AOM models of chinchilla with *S. pneumoniae, H. influenzae* type b, NTHi strain 86-028NP, and other ME-associated aerobic and anaerobic microbes have been developed through trans-bullar inoculation into the ME (Fulghum et al., 1982; Morton et al., 2012; Guan et al., 2014). Cholesteatomatous chronic OM chinchilla models have also been developed through the use of propylene glycol injection (Masaki et al., 1988).

#### 3.2.3 Mongolian gerbil

Mongolian gerbils have been widely used to establish AOM models for the study of AOM and analysis of efficacies of different antimicrobials. The small size and cost-effectiveness of Mongolian gerbils made them popular in OM studies. They have a reasonably large ME making it easy to inoculate through the overlying skin. It is also easy to sample ME fluid, however, they have narrow external ear canals. Mongolian gerbils normally have healthy ears and rarely experience OM, while cholesteatoma is common in elderly gerbils (Chole et al., 1981).

Compared to chinchillas and rats, fewer studies using gerbils as a model for bacterially induced AOM are reported. Direct bacterial ME inoculation has been used with *S. pneumoniae* type 3*, Haemophilus influenzae* type b, and NTHi strain 119 (Fulghum et al., 1982, 1985; Von Unge et al., 1997; Cenjor et al., 1998; Larsson et al., 2003; Unge et al., 2009). For non-bacterial ME disease, cholesteatoma development or other ME disease was reported in gerbils through ligation of the external ear canal, ET blocking by electrocauterization or glue, and chemical injections into the bulla (Larsson et al., 2005; Yamamoto-Fukuda et al., 2010, 2011).

#### 3.2.4 Guinea pig

The MEs of guinea pigs have structural similarities to those of mice and rats, but the size of bulla in this animal is smaller compared to gerbils and chinchillas (Hellström et al., 1982; Foxwell et al., 1998). Several studies have used the AOM guinea pig model developed by using non-typeable *S. pneumoniae* and *S. pneumoniae* type 3 (Sp3) via a trans-bullar approach (Parks et al., 1996; Dai et al., 2009; Guan and Gan, 2013). LPS from *Klebsiella pneumoniae* was injected into the ME of the guinea pig to develop an OME guinea pig model (Ohashi et al., 1991; Dai and Gan, 2008). Yu et al. developed a guinea pig model of OME by reversible ET obstruction (Yu et al., 2013). Cholesteatoma guinea pig models with chemical inoculants in the bulla have also been developed (Yamamoto-Fukuda et al., 2011).

#### 3.2.5 Monkey

The NP-ET-ME complex of primates has been employed to model the normal and pathological activities of the human ET. There have been very few studies on monkey models of OM. The Rhesus monkey model has been used to evaluate the impact of allergic rhinitis in the development of ET obstruction and OME (Friedman et al., 1981; Doyle et al., 1985).

Dohar et al. (1998, 2005) established a CSOM cynomolgus monkey model by infection of *P. aeruginosa* to investigate the safety and efficacy profile of topical ciprofloxacin hydrochloride for treating experimental CSOM. The tympanic membrane of cynomolgus monkeys was perforated and the ME was injected with a strain of *P. aeruginosa* which forms biofilms (Dohar et al., 1998, 2005). Although monkeys share more similarities to humans than rodents, their large size makes their handling in experimental settings very difficult. They are costly and not readily available, and modern ethical considerations have also reduced their use.

#### 3.2.6 Mouse models of OM

Since 2010, mouse OM models have significantly increased as compared to other models and now the mouse is more utilized in OM research because it offers many remarkable advantages over other animals [see review by (Bhutta, 2012)]. The wide availability of reagents allows complex analysis of immunological progression and host responses. Importantly, several genetic, transgenic, and gene deletion strains are available that can be used in studying various aspects of OM pathophysiology as well as host susceptibility to OM (Mitchell et al., 1997; Hardisty et al., 2003; MacArthur et al., 2006; Zheng et al., 2006).

Although mouse models offer many benefits as model systems for OM, yet there are some limitations to consider. The small size of the tympanic membrane in mice makes it difficult to access as compared to larger rodents. Even with the use of a microscope, surgical procedures on mice can be challenging. Inoculating and removing fluid from the mouse ME is difficult, and sampling large amounts of effusion is impractical because of the small size of the ME. The mouse is less hardy in tolerating general anaesthesia and surgical bleeding. Since major pathogens of OM such as NTHi, *S. pneumoniae,* and Mcat are not natural murine pathogens (Murphy, 2005; Doherty et al., 2006), variations can be observed in patterns of colonization and immune responses resulting from infecting mouse ME and NP than those observed in humans. This notable limitation is also associated with other rodent models. Nonetheless, it can be overcome via direct ME inoculation or multiple IN inoculations. Genetically modified mice which are more susceptible to human pathogens can be used for this purpose. Moreover, the use of mutant bacterial strains capable of adhering and invading the murine mucosa more effectively than wild-type bacteria offers another approach (Tu et al., 2007).

To study the natural course of OM in mice, Dewan et al. have described a novel pathogen capable of transmission between mice that causes OM (Dewan et al., 2019; Ma et al., 2022). *Bordetella pseudohinzii* efficiently replicates in the NP, quickly escalates the ET, and colonizes the ME. The resulting acute and chronic histopathological transformations with an increasing decline in hearing acuity closely represent OM in humans. Laboratory mice experimentally inoculated with a very small inoculum of *B. pseudohinzii* consistently had their MEs colonized and subsequently transferred it to cage mates (Dewan et al., 2019; Ma et al., 2022).

##### 3.2.6.1 Genetic mouse models of OM

Several mouse mutations from the ethylnitrosourea (ENU) mutagenesis program have been described that develop spontaneous OM. The deaf mouse mutant Jeff (Jf) is a single locus OM model that was discovered from the ENU program. A notable conductive hearing loss along with pus and fluid in the ME cavity is observed in heterozygous Jeff mouse postnatal day 35 and it develops a CSOM with a high rate of inflammation. Jeff mouse has a mutation in an F-box gene (FBXO11). The expression of this gene occurs in the epithelial cells of the ME from the latter stages of embryonic development until day 13 after birth (Hardisty et al., 2003). Homozygous Jeff mouse, on the other hand, demonstrates facial clefting, cleft palate, and perinatal death (Hardisty-Hughes et al., 2006). A study genotyped 13 single nucleotide polymorphisms (SNPs) in FBXO11 establishing the link between FBXO11 polymorphisms and chronic OM with effusion/recurrent OM (COME/ROM) (Segade et al., 2006). The Junbo (Jbo) mouse, again from the ENU mutagenesis program, has a mutation in the inflammatory-signaling regulator Evi1 (Parkinson et al., 2006). The heterozygote develops spontaneous CSOM. Another important mouse model from the ENU program is a genetic OM-one (gom1) mutant mouse. This mouse is prone to develop OME and develops many characteristics of OM such as craniofacial abnormalities, ME effusion, epithelial hyperplasia, and hearing loss. Thus, the gom1 mouse provides a remarkable tool for elucidating OM pathogenesis, presenting similar pathological changes and auditory dysfunction as those observed in human OM patients (Zheng et al., 2022). Gene targeting and other transgenic modifications have been used to develop genetic mouse models to investigate OM susceptibility in human patients with genetic defects. Patients having velo-cardio-facial syndrome/DiGeorge syndrome (VCFS/DGS) with 22q11 deletions commonly develop chronic OM (Funke et al., 2001). In bacterial artificial chromosome (BAC) transgenic mice, overexpression of the TBX1 transcription factor (an equivalent of human VCFS/DGS in mice) and three other transgenes resulted in similar defects as seen in VCFS/DGS patients (Liao et al., 2004). Mice with a deletion in the p73 locus (p73−/− mice) exhibited a 100% occurrence of OM (Yang et al., 2000). Deficiency of lymphocyte function-associated antigen-1 (LFA-1^−/−^ (CD11a/CD18)), in mice resulted in an elevated OM rate along with a notably high mortality rate (Prince et al., 2001). A dysmorphology of ET and abnormal ME cavity is observed in eya4 knockout mice leading to the development of OME (Depreux et al., 2008). A point mutation in the nischarin protein causes chronic OM resulting in conductive hearing loss development (Crompton et al., 2017). The mutant mouse strain known as ages-with-stiffened-joints (asj), carrying a point mutation in the Enpp1 gene, exhibits early-onset conductive hearing loss and defects in the ME with approximately 90% of the mutant mice have hearing loss (Tian et al., 2016). Furthermore, mutations in the EDA, EDAR, and EDARADD genes have been linked with the onset of nasopharyngitis, rhinitis, and OM (Azar et al., 2016).

In addition to investigating spontaneous OM in genetic mouse trains, several studies have investigated the known human otopathogens in these strains of mice. A high susceptibility to ME infections with *S. pneumoniae* was seen in lysozyme knockout mice (M^−/−^) mice leading to more pronounced inflammation in ME compared to wild-type mice (Shimada et al., 2008). Junbo heterozygote mice had NTHi intranasally inoculated, resulting in NTHi ascending and establishing in the ME (Hood et al., 2016). This mouse strain was also used to study vaccine responses (Hood et al., 2016) and cellular immune responses to NTHi OME (Vikhe et al., 2019). Kurabi et al. used Asc^−/−^ knockout mice to develop an OM model using NTHi strain 3,655. A comparison of responses to NTHi in the ME between wild-type and Asc^−/−^ mice showed persistent inflammation and delayed clearing of NTHi from the ME cavity of the latter (Kurabi et al., 2015). To define the function of CCL3 (a potent effector of inflammation) in OM, *ccl3*^−/−^ mice were infected with NTHi to induce OM. The *ccl3*^−/−^ mouse had prolonged mucosal hyperplasia and impaired bacterial elimination (Deniffel et al., 2017). Moreover, a genetic mouse model having a mutation in a G protein-coupled receptor was demonstrated to develop spontaneous OME (Kerschner et al., 2013). To date, there are no reports of Mcat establishing OM in these mice. Although great leaps forward have been made in the development of genetic mouse models of OM, nonetheless, more genetic OM models are needed to improve our grasp on genome-related interactions between hosts and pathogens to aid in the discovery of innovative therapies for better and alternative treatment approaches for human OM conditions.

##### 3.2.6.2 Humanized mouse model

The “humanized” mouse has become an invaluable tool for studying the human immune system in recent years. Son et al. developed the first humanized mouse model to study OM which had engraftment of CD34^+^ hematopoietic stem cells from human fetal liver and recapitulated the acute OM process utilizing NTHi. This model mimics the inflammatory responses of the ME to bacterial infection, immune cells’ recruitment, and typical recovery process thus allowing researchers to investigate human immunity in OM in preclinical settings (Son et al., 2022).

#### 3.2.7 Mcat model

A major challenge to Mcat vaccine development is the unavailability of a suitable animal model and a dependable correlate of protection. Under experimental settings, Mcat does not readily survive and replicate in the rodent NP. Although, chinchilla has been used in evaluating the NTHi and *S. pneumoniae* vaccine antigens, however, it readily clears Mcat from the ME (Bakaletz, 2009). Therefore, either NTHi and/or viruses are used in chinchillas to study Mcat infections and vaccine antigens (Armbruster et al., 2010; Brockson et al., 2012; Shaffer et al., 2013; Perez et al., 2014), or direct intra-bullae inoculation (Blakeway et al., 2019). Nevertheless, the chinchilla NP colonization model, the mouse lung clearance model, or the *in vitro* functional assays are unable to provide a fixed correlate of protection for assessment in animal models. Therefore, the development of improved animal models for evaluating potential Mcat vaccines is highly desired.

## 4 Concluding remarks

Vaccines are required to lower the burden of childhood OM disease worldwide and would also reduce the burden of bacterial diseases caused by these pathogens in other susceptible populations. Given the fact that OM is a polymicrobial disease, it presents numerous challenges for vaccine development. It would be most advantageous to develop an OM vaccine that targets all three causative bacteria to confer better protection against the disease. Progress has been made in identifying and investigating vaccine targets against OM, and several candidates from three major pathogens of OM are in different stages of development as vaccine antigens. Moreover, many of these antigens are ready for preclinical and early clinical testing. Investment in these studies will enable valuable advances in the development of new vaccines with better efficacy and coverage. Nonetheless, further research is required to discover and analyze new vaccine antigens. A crucial aspect of developing antigen-based strategies is to use highly conserved antigens that are shared between similar bacterial species. The combination of reverse vaccinology and immuno-informatics is increasingly being adopted in the search for new vaccine candidates. The combination of these two approaches has the potential to reduce both time and costs before taking a vaccine into clinical trials thus providing a better estimate of how human populations would respond to the vaccine candidate worldwide.

Several animal models of OM are available. Researchers studying OM should be familiar with the strengths and limitations of these models to select the model that best fits their experimental needs. While chinchillas and rats have been favored animals to date for OM research, recently, many mouse models have been developed through NP colonization and ME infection. Many groups have used these models to demonstrate induction of protection against experimental OM and NP carriage following IN vaccine administration. Knockout and transgenic mice have proved to be vital for revealing the roles of different components of a host immune response relevant to susceptibility. Furthermore, the humanized mouse model offers the potential to execute a range of studies on human immunity in OM. However, there is still a need to develop an appropriate OM model that will allow rapid and high throughput assessment of Mcat vaccine candidates in preventing disease. Moreover, a mouse model of OM developed using polymicrobial infection (NTHi, *S. pneumoniae*, and Mcat) would allow the assessment of polymicrobial OM vaccines. Until we have a vaccine effective against the three major otopathogens, the global burden of acute and chronic OM morbidity will continue.

## Author contributions

AZ: Conceptualization, Writing – original draft, Writing – review & editing. JW: Supervision, Writing – review & editing. IG: Supervision, Writing – review & editing. IP: Supervision, Writing – review & editing.
